# A novel prognostic model for prostate cancer based on androgen biosynthetic and catabolic pathways

**DOI:** 10.3389/fonc.2022.950094

**Published:** 2022-11-10

**Authors:** Aoyu Fan, Yunyan Zhang, Jiangting Cheng, Yunpeng Li, Wei Chen

**Affiliations:** Department of Urology, Zhongshan Hospital, Fudan University, Shanghai, China

**Keywords:** androgen biosynthesis, androgen catabolism, consensus, gene signature, survival analysis, prostate cancer, androgen metabolism

## Abstract

Prostate cancer (PCa) is one of the most common malignancies in males globally, and its pathogenesis is significantly related to androgen. As one of the important treatments for prostate cancer, androgen deprivation therapy (ADT) inhibits tumor proliferation by controlling androgen levels, either surgically or pharmacologically. However, patients treated with ADT inevitably develop biochemical recurrence and advance to castration-resistant prostate cancer which has been reported to be associated with androgen biosynthetic and catabolic pathways. Thus, gene expression profiles and clinical information of PCa patients were collected from TCGA, MSKCC, and GEO databases for consensus clustering based on androgen biosynthetic and catabolic pathways. Subsequently, a novel prognostic model containing 13 genes (*AFF3*, *B4GALNT4*, *CD38*, *CHRNA2*, *CST2*, *ADGRF5*, *KLK14*, *LRRC31*, *MT1F*, *MT1G*, *SFTPA2*, *SLC7A4*, *TDRD1*) was constructed by univariate cox regression, lasso regression, and multivariate cox regression. Patients were divided into two groups based on their risk scores: high risk (HS) and low risk (LS), and survival analysis was used to determine the difference in biochemical recurrence-free time between the two. The results were validated on the MSKCC dataset and the GEO dataset. Functional enrichment analysis revealed some pivotal pathways that may have an impact on the prognosis of patients including the CDK-RB-E2F axis, G2M checkpoint, and KRAS signaling. In addition, somatic mutation, immune infiltration, and drug sensitivity analyses were performed to further explore the characteristics of HS and LS groups. Besides, two potential therapeutic targets, *BIRC5* and *RHOC*, were identified by us in prostate cancer. These results indicate that the prognostic model may serve as a predictive tool to guide clinical treatment and provide new insight into the basic research in prostate cancer.

## Introduction

Prostate cancer is the second most common cancer and the leading cause of cancer-related deaths in males, the detection rate of which in developing countries is increasing annually (1). After early diagnosis, patients can benefit from radical prostatectomy and androgen deprivation therapy (2, 3), but the biochemical recurrence and further tumor progression to advanced prostate cancer and castration-resistant prostate cancer (CRPC) still occur in many patients (4). In general, the treatment of advanced prostate cancer remains challenging.

The Androgen receptor plays an indelible role in the development and progression of prostate cancer, therefore, targeting androgen metabolism and androgen receptor are always the theme of treatment (5). For instance, leuprolide and goserelin inhibit androgen production by targeting gonadotrophin-releasing hormones. Besides, abiraterone, a CY17 inhibitor, further decreases androgen levels by reducing androgen production in non-gonadal tissues (6). The androgen biosynthetic and catabolic pathways have been shown to be associated with prostate cancer progression, which may be related to the different sensitivity of patients to ADT (7). Therefore, investigating the relevance of the androgen biosynthetic and catabolic pathways to prostate cancer progression is the focus of this research.

In this study, we constructed a prognostic model related to androgen biosynthetic and catabolic pathways and investigated multi-omics differences between high risk (HS) and low risk (LS) populations through comprehensive bioinformatics analysis. In conclusion, the novel risk model demonstrated excellent prognostic ability and may be beneficial in clinical treatment.

## Materials and methods

### Data set identification and preparation

498 PRAD (Prostate adenocarcinoma) gene expression data (FPKM form) was downloaded from the TCGA website (https://portal.gdc.cancer.gov/repository). The Memorial Sloan Kettering Cancer Center (MSKCC) and GSE70770 cohort downloaded from the cBioPortal database (8) (https://www.cbioportal.org/) and GEO database (https://www.ncbi.nlm.nih/geo/query/), respectively. 140 and 111 samples with complete clinical information in MSKCC and GSE70770 cohorts were selected as the validation set.

### Consensus clustering and differentially expressed genes analysis

A total of 13 genes (*HSD3B1*, *HSD17B6*, *SRD5A2*, *SRD5A3*, *CYP17A1*, *HSD17B3*, *SRD5A1*, *CYP19A1*, *HSD17B11*, and *HSD3B2*) in androgen biosynthetic and catabolic pathways (ABCGs) were collected from MsigDb (http://www.gsea-msigdb.org/gsea/msigdb/genesets.jsp). Subsequently, we constructed a (protein-protein interaction) PPI network in STRING (https://cn.string-db.org, version 11.0b) to elucidate the interactions of these 13 ABCGs. We used an unsupervised method based on Euclidean distance and Ward’s linkage and the optimal clustering number was determined according to the data difference percentage by the ConsensusClusterPlus package (9) with 1000 repeats. Through the implementation of the Benjamini and Hochberg (BH) method to compute gene expression changes, the R package limma (10) was used to identify the DEGs between the two clusters. The cutoff criteria were set as |log2FC| > 0.6 while P-Value < 0.05 for differential expression.

### Construction and assessment of the prognostic prediction model

Recurrence-free survival-related genes were screened by Univariate Cox regression. To reduce the risk of overfitting, the least absolute shrinkage and selection operator (LASSO) regression was performed. Then multivariate Cox regression was used to establish the prognostic prediction model. The risk score was calculated by (expression of gene1 × coefficient of gene1) + (expression of gene 2 ×coefficient of gene 2) + ⋯ + (expression of gene 13 × coefficient of gene 13). Principal component analysis (PCA) by UMAP function was performed on HIPLOT web tools (https://hiplot.com.cn/) for dimensionality reduction analysis between the two risk groups. The patients were divided into the high score (HS) and low score (LS) groups according to the optimal cutoff value by the survminer R package (Cutoff value=0.6855398). Kaplan-Meier(K-M) survival analysis and log-rank test were exploited to demonstrate the difference between the two groups. Single-gene survival curves for 13 genes in the prognostic model were analyzed on GEPIA web tools (http://gepia.cancer-pku.cn/). Subsequently, we further evaluate the predictive accuracy of the gene signature using the time-dependent receiver operating characteristic (ROC) curve by pROC R package. The area under the curve (AUC) was used to measure the discrimination power of the model. Calibration plots were performed to depict the prognostic predictive accuracy of the nomogram by RMS R package.

### Functional enrichment analysis and ceRNA network construction

Gene set enrichment analysis (GSEA) (11) was performed for the protein-coding genes. We downloaded 50 hallmark gene sets and 7 androgen-related gene sets from MsigDb (http://www.gsea-msigdb.org/gsea/msigdb/genesets.jsp). Subsequently, gene set variation analysis (GSVA) (12) was conducted to evaluate the differences in the activity of tumor hallmark gene sets and androgen-related gene sets between HS and LS groups.

To construct the competing endogenous RNAs (ceRNA) network, we analyzed the differentially expressed long non-coding RNAs (lncRNAs), miRNAs, and genes between the two risk groups with limma R package (|Log2FC| >0.5 and p-value <0.05), and subsequently predicted the targets of differentially expressed lncRNA and miRNAs using miRWalk (http://mirwalk.umm.uni-heidelberg.de) and miRcode (http://www.mircode.org) web tools. Finally, a ceRNA network based on the genes in the model was created after the match of differentially expressed lncRNAs, miRNAs, and genes.

### Somatic mutation distribution and characteristics of immune infiltration

The landscape of somatic mutations was depicted between the two groups and differentially mutated genes were detected by the maftools R package. The immune infiltration evaluation was achieved by using the IOBR R package (13). THE IOBR R package is a powerful and comprehensive immuno-oncology analysis tool. CIBERSORT, xCell, and EPIC are frequently-used open-source deconvolution methodologies in the IOBR R package. CIBERSORT, as the most popular deconvolution method, can detect 22 immune cells in the tumor microenvironment. xCell method can analyze the infiltration of 64 immune cells based on RNA-seq data. EPIC processes gene expression according to the immune cell phenotype to predict the cell subpopulation landscape. In addition, we evaluated the expression of 45 immune checkpoints and visualized the differences between the two groups using the ggplot2 R package.

### Drug sensitivity analysis and target prediction

Estimated IC50 of commonly used drugs for prostate cancer (PCa) patients in the TCGA dataset were calculated using the Genomics of Drug Sensitivity in Cancer (GDSC, https://www.cancerrxgene.org) and the Cancer Therapeutics Response Portal (https://portals.broadinstitute.org/ctrp) *via* oncoPredict R package. Human cancer cell lines (CCLs) expression profile data were collected from the Broad Institute-Cancer Cell Line Encyclopedia (CCLE) project (https://portals.broadinstitute.org/ccle/) (14). The CERES scores of 739 cell lines were obtained from the dependency map (DepMap) portal (https://depmap.org/portal/), with the lower the CERES score, the more important the gene was to cancer cell growth and survival.

### Statistical analysis

All analyses were performed in R software (version 4.1.1). The log-rank test was applied to evaluate the difference in higher recurrence-free survival (RFS) between the two groups in the Kaplan-Meier survival analysis. And in the comparison of differences between groups in clinical phenotype, immune infiltration, and drug sensitivity, either Student’s t-test was used if the variable was normally distributed or Wilcoxon rank-sum test was used. The correlation between two continuous variables was measured using Spearman’s rank-order correlation. The tests in this study were two-sided and the significance threshold was set as 0.05 except for univariate cox regression (p<0.2).

## Results

### Consensus clustering cased on ABCGs and identification of DEGs

**Figure 1:**
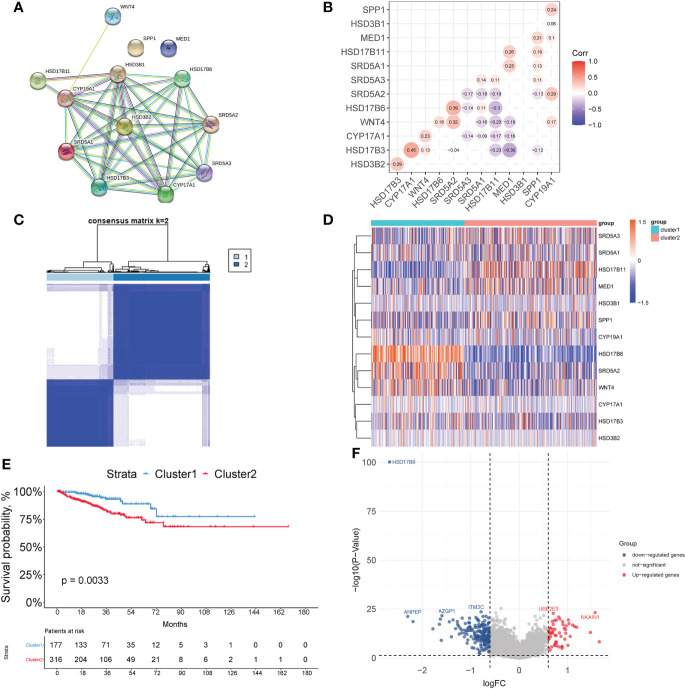
Consensus clustering based on androgen biosynthetic and catabolic pathways. **(A)** The PPI network of ABCGs constructed in the STRING database. **(B)** Gene expression correlation of ABCGs in TCGA cohort. **(C)** Consensus Clustering matrix (k =2). **(D)** Heatmap of ABCGs in two clusters. **(E)** Kaplan-Meier curves of RFS. **(F)** The different expression genes (DEGs) between cluster1 and cluster 2.

*HSD3B1*, *HSD17B6*, *SRD5A2*, *SRD5A3*, *CYP17A1*, *HSD17B3*, *SRD5A1*, *CYP19A1*, *HSD17B11*, and *HSD3B2* have a strong link in the PPI network as shown in **Figure 1A**. Furthermore, the expression correlations of these genes are highly significant (**Figure 1B**). By using an unsupervised method based on Euclidean distance and Ward’s linkage, the optimal number of clusters was determined based on the percentage difference in data from 1000 iterations, 498 patients were classified into two clusters (**Figure 1C**). The heat map showed that these genes are significantly differentially expressed in the two clusters and the Kaplan-Meier curve showed cluster1 had a better prognosis compared to cluster2 (p=0.0033) (**Figures 1D, E**). According to the expression heatmap of these 13 original ABCGs in the TCGA PRAD database, we found that WNT4, HSD17B6, and SRD5A2 were highly expressed in cluster1, and SRD5A3, HSD17B11, MED1, and SPP1 were highly expressed in cluster2 (**Figure 1D**). Subsequently, 242 DEGs were screened, including 57 up-regulated genes and 185 down-regulated genes. The top five genes that were significantly up-regulated in cluster2 were *CRISP3*, *NKAIN1*, *ERG*, *F5*, and *LRRN1*. Besides, *HSD17B6*, *ANPEP*, *ALOX15B*, *TFF3*, and *MT1G* were the top five down-regulated genes (**Figure 1F**). Overall, the above results indicated that the ABCGs had an impact on the prognosis of patients.

### Construction and validation of the prognostic model

**Figure 2:**
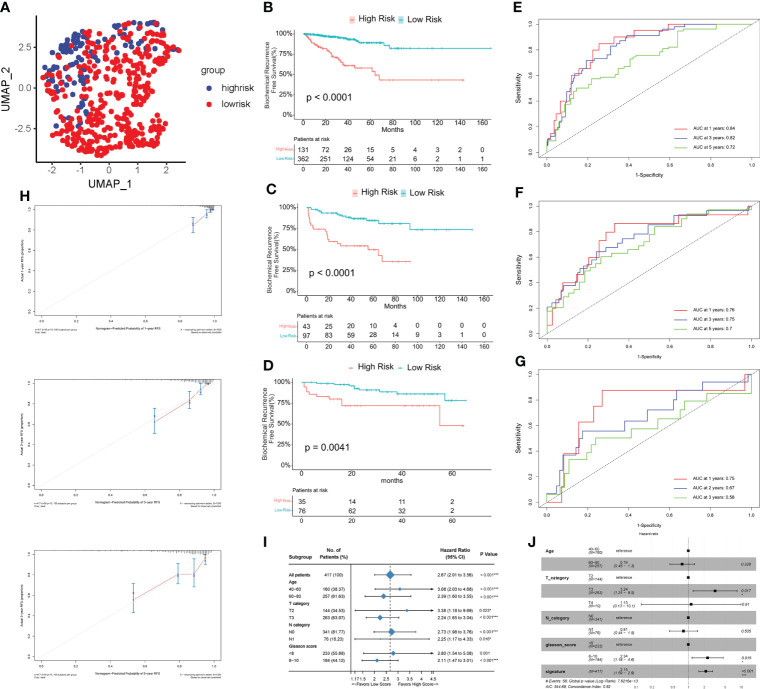
Assessment of the risk model. **(A)** Principal component analysis (PCA) of TCGA PRAD cohort. **(B–D)** Kaplan-Meier curves of RFS between high risk (HS) and low risk (LS) groups in TCGA **(B)**, MSKCC **(C)**, and GSE70770 **(D)** cohort. **(E–G)** Time-dependent receiver operating characteristic (ROC) analysis in TCGA **(E)**, MSKCC **(F)**, and GSE70770 **(G)** cohort. **(H)** Calibration plots for the nomogram: 1-, 3-, and 5- year nomogram. **(I)** Clinical subgroup analysis. **(J)** Univariate cox regression analysis of the clinical features and gene signature. (*P < 0.05, ***P < 0.001).

A total of 149 genes were identified as being associated with time to biochemical-free relapse from 242 genes in TCGA samples using univariate cox regression. Ultimately, a prognostic model for prostate cancer was established by lasso-cox regression (**Supplementary Figures 1A, B**). The prognostic model includes 13 genes (*AFF3*, *B4GALNT4*, *CD38*, *CHRNA2*, *CST2*, *ADGRF5*, *KLK14*, *LRRC31*, *MT1F*, *MT1G*, *SFTPA2*, *SLC7A4*, *TDRD1*) and the risk score = (-0.1028 * Exp*AFF3*) + (0.2922 * Exp*B4GALNT4*) + (-0.511 * Exp*CD38*) + (0.0072 * Exp*CHRNA2*) + (0.038318 * Exp*CST2*) + (0.2041 * Exp*ADGRF5*) + (0.1326 * Exp*KLK14*) + (0.1493 * Exp*LRRC31*) + (-0.1262 * Exp*MT1F*) + (-0.0116 * Exp*MT1G*) + (-0.1639 * Exp*SFTPA2*) + (-0.1697 * Exp*SLC7A4*) + (-0.4706 * Exp*TDRD1*) (**Supplementary Figure 1C**). After performing survival analysis of these gene expressions with recurrence-free survival, we found that the expression of AFF3, CD38, MT1F, MT1G, SFTPA2, and SLC7A4 positively correlated with recurrence-free survival, and the expression of B4GALNT4 was negatively correlated with recurrence-free survival (p<0.05). Their coefficients are consistent with their responsiveness to androgen (**Supplementary Figure 2**). Based on the optimal cutoff value, the samples were divided into HS and LS groups (Cutoff value=0.6855398). Principal component analysis (PCA) shows that people in the HS group are distributed in different directions from those in the LS group (**Figure 2A**). **Figure 2B** shows that the LS group had significantly RFS than the HS group (p<0.0001), demonstrating that a higher score indicates a worse prognosis and increased risk. To evaluate the clinical features of the HS and LS groups, race, age, TNM stage, and Gleason score were compared between the two groups (**Table 1**). The results showed that the TNM stage and Gleason scores were higher in the HS group. Subsequently, the predictive performance of the prognostic risk model was evaluated by ROC curves with AUC of 0.84, 0.82, and 0.72 for 1, 3, and 5 years, respectively (**Figure 2E**). Then the predictive capability of the prognostic model was validated in MSKCC (Cutoff value=-1.790379) (p<0.0001), and GSE70770 (Cutoff value=-2.053357) (p=0.0041), all of which showed the risk model was negatively correlated with RFS (**Figures 2C, D**). The AUC was also assessed in the MSKCC dataset and GSE70770 dataset (**Figures 2F, G**). A nomogram of the risk model was created (**Supplementary Figure 1D**) and the calibration plots revealed remarkable accuracy in predicting biochemical recurrence (**Figure 2H**). Subsequently, a clinical subgroup analysis was conducted to verify the validity of the prognostic model in various clinical subgroups. Whether stratified by age, T-stage, N-stage, or Gleason score, the results indicated that the risk score was a danger factor for RFS in each clinical subgroup (**Figure 2I**). The forest plot illustrates the relationship between RFS and these clinical phenotypes, from which we could see that age, TNM stage (M-stage was omitted because of the scarcity of M1 patients), Gleason score, and risk score were negatively implicated in RFS (**Figure 2J**).

**Table 1:** Clinical features of TCGA cohort.

	High risk	Low risk	P value
	*N=131*	*N=362*	
**Race**			0.467
asian	4 (3.05%)	8 (2.21%)	
black	11 (8.40%)	46 (12.7%)	
white	111 (84.7%)	298 (82.3%)	
not reported	5 (3.82%)	10 (2.76%)	
**Age**	62 [58;67]	61 [56;66]	0.034
**T**			<0.001
T2	25 (19.1%)	163 (45.0%)	
T3	100 (76.3%)	189 (52.2%)	
T4	4 (3.05%)	6 (1.66%)	
not reported	2 (1.53%)	4 (1.10%)	
**N**			<0.001
N0	81 (61.8%)	264 (72.9%)	
N1	37 (28.2%)	39 (10.8%)	
not reported	13 (9.92%)	59 (16.3%)	
**M**			0.118
M0	118 (90.1%)	332 (91.7%)	
M1	2 (1.53%)	0 (0.00%)	
not reported	11 (8.40%)	30 (8.29%)	
**Gleason score**			<0.001
6-7	39 (29.8%)	256 (70.7%)	
8-9	92 (70.2%)	106 (29.3%)	

### Functional enrichment analysis and ceRNA network

**Figure 3:**
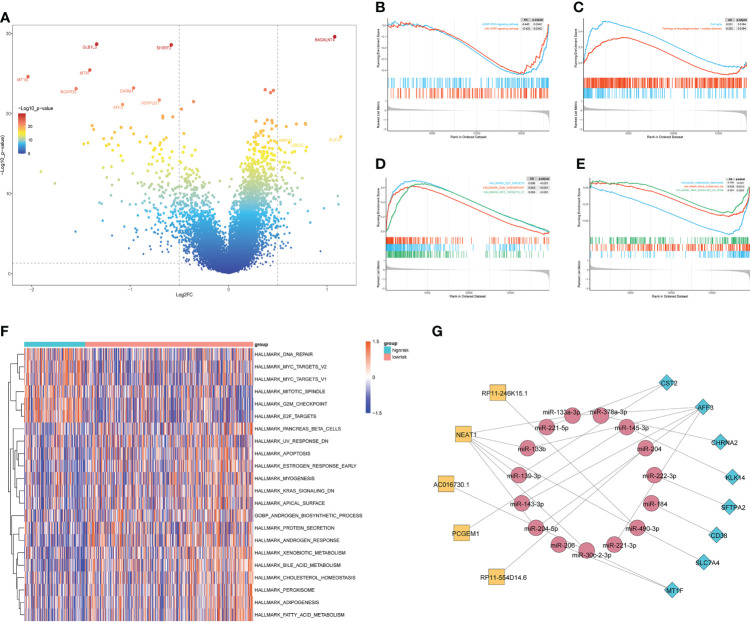
The enrichment of signaling pathways in HS and LS groups. **(A)** The DEGs between the HS and LS groups. **(B–E)** Gene Set Enrichment Analysis (GSEA) of TCGA cohort to identify signaling pathways associated with the risk model. **(F)** Enrichment scores of Gene Set Enrichment Analysis (GSVA) among 22 gene sets which are significant differences between the HS and LS groups. **(G)** Competing endogenous RNAs (ceRNA) network based on the genes in the risk model.

To investigate the biological processes associated with the difference in relapse-free survival between the two groups, we performed a functional enrichment analysis of DEGs between the HS and LS groups. 285 DEGs were identified, including 73 upregulated and 212 downregulated ones (**Figure 3A**). Subsequently, we performed a GSEA analysis and the results showed that the cGMP-PKG signaling pathway, JAK-STAT signaling pathway, cell cycle, and neurodegeneration-multiple disease signaling differed between HS and LS groups (**Figures 3B, C**). Moreover, E2F targets, G2M checkpoint, MYC targets, androgen response, KRAS signaling, and TNFA *via* NF-κB signaling were among the signaling pathways differently enriched between the two groups (**Figures 3D, E**). Subsequently, we explored the enrichment of two groups in 50 signaling pathways and 7 androgen-related pathways with GSVA. The results showed that the 22 gene sets had statistically significant differences in GSVA enrichment scores between the two groups (**Figure 3F**). These results may help us to discover the critical pathways associated with this risk model. Finally, we constructed a ceRNA network containing 5 lncRNAs, 15 miRNAs, and 8 mRNAs based on genes in the risk model to further understand how lncRNAs regulate mRNA expression through sponging miRNAs. (**Figure 3G**). As an important lncRNA, *NEAT1* may be involved in regulating six miRNAs in the ceRNA network and indirectly affect the expression of genes in the model.

### Differential analysis of somatic mutations

**Figure 4:**
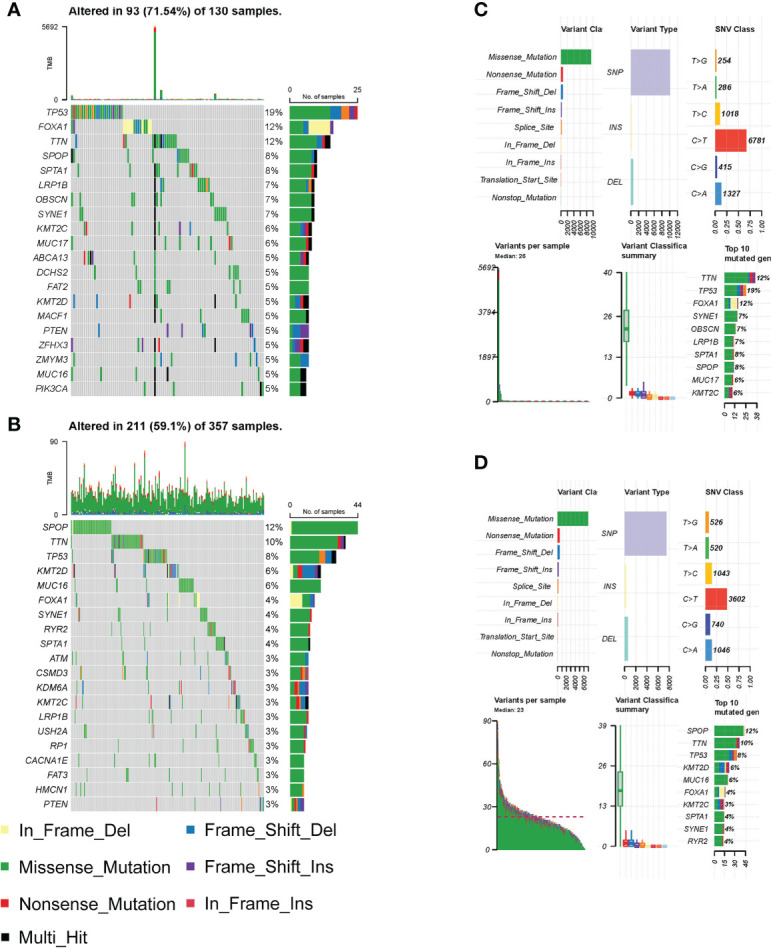
Differential analysis of somatic mutations. **(A–B)** The top 20 highly mutated genes in HS **(A)** and LS **(B)** groups. **(C–D)** Detailed gene mutation summary in HS **(C)** and LS **(D)** groups.

Genetic mutations are associated with patient prognosis in many malignancies (15). We consequently explored the differences in genetic alterations in tumors in HS and LS groups. **Figures 4A, B** showed the landscape of the top 20 highly mutated genes in the two groups. The results revealed that in the HS group, *TP53*, *FOXA1*, and *TTN* had the highest mutation frequencies of 19%, 12%, and 12%, respectively (**Figure 4A**). As for the LS group, *SPOP*, *TTN* and *TP53* had the highest mutation frequencies of 12%, 10%, and 8%, respectively (**Figure 4B**). The difference in frequency of *TP53* and *FOXA1* mutations was quite substantial, implying that *TP53* and *FOXA1* mutations may have a role in patient prognosis. In the following, we summarized the detailed gene mutations in the HS and LS groups (**Figures 4C, D**).

### Characterization of immune cell infiltration in the tumor microenvironment

**Figure 5:**
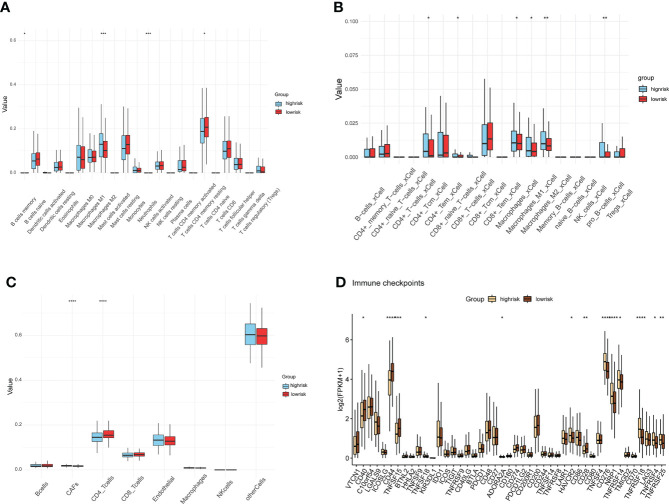
Differences in immune cell infiltration and immune checkpoints. **(A–C)** Assessment of tumor microenvironment and immune cell infiltration by methods CIBERSORT **(A)**, xCell **(B)**, and EPIC **(C)** in HS and LS groups. **(D)** differences analysis of immune checkpoints between HS and LS groups. (*P < 0.05, **P < 0.01, ***P < 0.001, ****P < 0.0001).

Ongoing research into the TME highlighted the important role that immune cell infiltration plays in tumor progression (16). We found a higher infiltration of Macrophage M2 and a lower infiltration of memory-resting CD4 T cell in the HS group compared to the LS group by the CIBERSORT method (**Figure 5A**). Results of the xCell method showed differential infiltration of CD4 Central Memory T (Tcm) cells, CD8 naive T cells, M1 macrophages, M2 macrophages, and pro B cells in the TME in two risk groups (**Figure 5B**). Subsequently, we used the EPIC method to examine the TME of the two groups separately and 7 cell types were assessed. Of these, cancer-associated fibroblasts (CAFs) had a higher proportion, while CD4 T cells had a lower infiltration in the HS group (**Figure 5C**). The differences in the expression of the common immune checkpoints between the two groups indicated that several immune checkpoints were highly expressed in the HS group, including *TNFSF18*, *ADORA2A*, *HAVCR2*, *CD28*, *CD276*, *NRP1*, *TNFRSF14*, *TNFRSF18*, *TNFRSF4*, *TNFRSF25* (**Figure 5D**). These immune checkpoints, which are prominently expressed in the HS group, may be potential targets for immunotherapy in prostate cancer.

### Drug sensitivity analysis and target prediction

**Figure 6:**
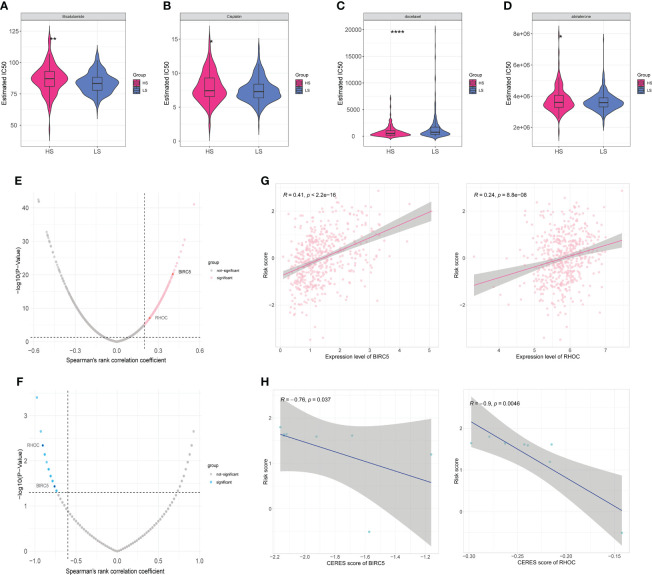
Drug sensitivity and identification of potential drug targets. **(A–D)** Estimated IC50 indicates the efficiency of four common drugs in prostate cancer (Bicalutamide **(A)**, Cisplatin **(B)**, Docetaxel **(C)**, and Abiraterone **(D)**) in HS and LS groups. **(E)** Volcano plot of Spearman’s correlations and significance between risk score and expression of drug targets. Red dots indicate the significant positive correlations (Spearman’s r>0.2, p<0.05). **(F)** Volcano plot of Spearman’s correlations and significance between risk score and CERES score of drug targets. Blue dots indicate the significant negative correlations (Spearman’s r <−0.6, P <0.05). **(G)** Scatter plots of Spearman’s correlations and significance between risk score and expression of BIRC5 (left) and RHOC (right). **(H)** Scatter plots of Spearman’s correlations and significance between risk score and CERES score of BIRC5 (left) and RHOC (right). (*P < 0.05, **P < 0.01, ****P < 0.0001).

In order to guide clinical use based on the risk model, we predicted the IC50 of four drugs (Bicalutamide, Cisplatin, Docetaxel, and Abiraterone) for two risk groups with CTRP and GDSC databases, respectively. Bicalutamide, cisplatin, and abiraterone had greater IC50 values in the HS group (**Figures 6A, B, D**), indicating that patients in the HS group were less susceptible to these three medications than those in the LS group, but the sensitivity to docetaxel was higher in the HS group (**Figure 6C**). These findings revealed that drug sensitivity was one of the factors influencing the outcome of the two groups. Since drug target genes positively correlated with risk scores may have potential therapeutic significance, 2136 drug target proteins were collected for screening candidate targets. First, we calculated the correlation coefficients between drug target gene expression levels and risk score and identified 294 drug target genes that were positively associated with the risk score (Spearman’s r>0.2, p<0.05) (**Figure 6E**). Subsequently, we further identified 48 drug targets with a negative correlation (Spearman’s r <−0.6, P <0.05) between risk scores and CERES scores of prostate cancer cell lines (**Figure 6F**). Ultimately, four drug target proteins were matched, including *B4GALT4*, *BIRC5*, *RHOC*, and *SULT1E1*. They were highly expressed in the high score population and were important for the growth and survival of prostate cancer cells. Notably, the CERES scores of *B4GALT4* and *SULT1E1* were not less than zero in some prostate cancer cell lines, indicating that *B4GALT4* and *SULT1E1* might not play an inhibitory role in PRAD. Thus, the drugs targeting *BIRC5* and *RHOC* may be potential targets in the treatment of prostate cancer (**Figures 6G, H**).

## Discussion

Androgens and androgen receptor play a critical role in prostate cancer oncogenesis, and ADT has traditionally been an essential first-line treatment for PCa (3). However, almost all advanced prostate cancer patients experience a re-elevation of PSA after treatment with ADT and enter the phase of castration-resistant prostate cancer (3, 4). In this study, 498 prostate cancer patients in the TCGA database were divided into two clusters and 242 DEGs were screened. According to the expression heatmap of these 13 original ABCGs in the TCGA PRAD database (**Figure 1D**), WNT4, HSD17B6, and SRD5A2 were highly expressed in cluster1, and SRD5A3, HSD17B11, MED1, and SPP1 were highly expressed in the cluster2. Previous studies have shown that AR protein expression can be strongly suppressed by Wnt activation (17). The protein encoded by HSD17B6 has both oxidoreductase and epimerase activities and is involved in androgen metabolism (18). In contrast, SRD5A3 is involved in the production of the androgen 5-alpha-dihydrotestosterone (DHT) from testosterone and maintains the androgen and androgen receptor activation pathway (19). HSD17B11, MED1, and SPP1 are involved in androgen synthesis and AR receptor activation (20–22). This suggests that in cluster2, androgen synthesis and AR receptor activation may be more active. According to survival analysis, patients in cluster 2 have a worse prognosis, which could be attributed to androgen signaling activation (**Figure 1E**).

Numerous research has reported the 13 genes in the model. High AFF3 expression in ER+ breast cancer was linked to a poor overall survival rate, and upregulation of AFF3 underlies tamoxifen resistance in ER+ breast cancer (23). Interestingly, the effect of AFF3 on prostate cancer appears to be the inverse of that on ER+ breast cancer, implying that AFF3 in androgen and estrogen metabolism warrants additional exploration (**Supplementary Figure 2A**). B4GALNT4 is linked to malignant behavior and maybe a new prognostic marker for esophageal squamous cell carcinoma (24). The protein CD38 participates in the pathogenesis and regulation of metabolism in a variety of diseases, including obesity, diabetes, heart disease, asthma, and inflammation (25). Several CD38-targeting antibodies, daratumumab, isatuximab, and MOR202, have been developed for the treatment of multiple myeloma (26). The role of *CD38* in tumor progression has also been reported in prostate cancer, and multiple studies suggest that *CD38* could be a potential immunotherapy target (27). However, a recent phase I/II open-label, multicenter study observed a lack of efficacy of isatuximab (anti-CD38 monoclonal antibody) in metastatic castration-resistant prostate cancer (28). CHRNA2 was discovered to promote thermogenesis in uncoupling protein 1 (Ucp1)-positive beige adipocytes through a cAMP- and protein kinase A-dependent pathway. Further research on its role in prostate cancer and androgen metabolism is required (29). CST2 encodes a thiol protease inhibitor which was found to be associated with tumorigenesis as well as poor prognosis in breast and gastric cancers (30, 31). *ADGRF5* (*GPR116*), predicted to enable G protein-coupled receptor activity, has previously been shown to promote breast cancer metastasis (32). *KLK14* is a Protein Coding gene encoding a member of the kallikrein subfamily of serine proteases which has been reported to be associated with the progression of various cancers including prostate cancer and breast cancer (33–35). LRRC31 was found to act as a DNA repair inhibitor that sensitizes breast cancer brain metastasis to radiation which can be targeted for cancer radiosensitizing therapy (36). *MT1F* and *MT1G* belong to Metallothioneins (MTs), which enable zinc ion binding activity and involve in the cellular response to the metal ion, DNA damage, and oxidative stress. MTs also play a pivotal role in the progression and drug resistance of multiple tumors (37). Studies have shown that SFTPA2(Surfactant Protein A2) mutations are associated with interstitial lung disease and lung cancer, but its role in other tumors, including prostate cancer, requires further study (38). SLC7A4 is involved in amino acid transmembrane transport activity and has been shown to have a higher expression in melanoma tissues than in normal skin tissues, while the relatively high expression of SLC7A4 has a poorer prognosis in skin cutaneous melanoma (SKCM) patients, which indicates that it may serve as a novel tumor marker in SKCM (39). *TDRD1* has been discovered to be a direct target of (ETS-related gene) ERG, which promotes tumor initiation and progression in TMPRSS2-ERG fusion prostate cancer and could be a new immunotherapy target (40–42).

The construction of the risk model allowed for the accurate prediction of patient RFS and treatment guidance. The results of GSEA and GSVA revealed that the HS and LS groups differed in several signaling pathways (**Figures 5B–E**). In prostate cancer, MYC amplification, and TP53 mutation are common genetic changes (43). GSVA showed that the MYC targets were enriched in the HS group, and the mutation analysis revealed TP53 mutation rates of up to 19% in the HS group, compared to 8% in the LS group (**Figures 3A, B**, **5F**). Mutations in the tumor suppressor gene TP53 promote cancer growth in a variety of ways (44). TTN mutations cause changes in cell signaling pathways, the expression of immunological checkpoints, and immune cell infiltration (45). The TP53 mutation rate was higher in the HS group than in the LS group, which, on the one hand, reflects the relationship between differences in TP53 and TTN mutations in the two groups and the prognosis of prostate cancer, and on the other hand, whether the difference in TP53 and TTN mutations rate between the two groups of patients is related to androgen metabolism requires further study. Neuroendocrine prostate cancer accounts for a minimal number of pathological types of prostate cancer, but neuroendocrine differentiation plays an important role in the progression of drug resistance in some prostate cancer patients (46). GSEA illustrated that neurodegeneration-multiple disease signaling was enriched in the HS group, which further validated the impact of neuroendocrine differentiation on PCa prognosis and suggested the potential role of androgen metabolism on neuroendocrine differentiation.

The immune system has a dual role in cancer, as immune cells not only destroy cancer cells to inhibit tumor proliferation but also participate in the regulation of TME to speed up tumor progression (47). Several studies have shown that the infiltration level of tumor-associated macrophages correlates with tumor aggressiveness. After treatment with ADT, CD68+ and CD163+ macrophage infiltration was increased in the tumor tissues of patients (48). Hence, due to the differential infiltration of macrophages in the HS and LS groups (**Figures 5A–C**), which further suggests that androgen metabolism may play a role in the tumor microenvironment, the causal relationship needs to be further explored. All the above suggested that this risk model was related to tumor immune infiltration and provided a reference for personalized immunotherapy.

Bicalutamide is a nonsteroidal anti-androgen and abiraterone is a selective inhibitor of CYP17 to suppress androgen biosynthesis (49, 50). The sensitivity to abiraterone and bicalutamide was higher in the LS group compared to the HS group, and GSVA also showed that androgen response and biosynthetic processes were enriched in the LS group. Therefore, these 13 genes in the model may be correlated with drug sensitivity and have an impact on patient prognosis. However, the potential impact of androgen biosynthetic and catabolic pathways on these genes needs to be further investigated.

*RHOC*, encoding a member of the Rho family of small GTPases, acts as a molecular switch to regulate signal transduction pathways during the cell cycle and the formation of myosin contractile loops in the cytoplasmic division (**Figures 6G, H**). It has been shown to play an indispensable role in promoting the invasion and metastasis of breast, pancreatic, and lung cancers (51). A phase I/II clinical trial showed that a vaccine targeting *RHOC* was well tolerated and safe in prostate cancer patients, induced effective and durable T-cell immunity, and delayed tumor metastasis and recurrence (52). Animal models indicated that it is not necessary for embryogenesis (53), which opens up the possibility of serving as a tumor marker and therapeutic target.

There are some limitations to our study. First, the risk model needs to be further validated in a larger cohort as well as in a prospective study. Second, we only performed a rough study on the prognostic significance of androgen biosynthetic and catabolic pathways in prostate cancer, and its specific mechanisms were not explored. Third, we lacked data on patients with recurrence of 10 to 15 years and it is necessary to expand the sample of advanced nonlocalized prostate cancer. In addition, the findings for patients receiving local treatment were limited, and the clinical information on the samples needs to be expanded.

## Conclusion

Overall, we developed a prognostic model for prostate cancer based on androgen biosynthetic and catabolic pathways, and multi-omics analyses demonstrated that the signature was related to tumor mutations, immune infiltration, and drug sensitivity, which influenced prostate cancer prognosis. In-depth investigations of the genes in this model to explore their potential as tumor markers and therapeutic targets may be useful for our understanding of the molecular mechanisms of prostate cancer progression and recurrence.

## Data availability statement

The original contributions presented in the study are included in the article/**Supplementary Material**. Further inquiries can be directed to the corresponding author.

## Author contributions

Conceptualization was contributed by WC. Data collection and curation were contributed by AF and YL. Data analysis and interpretation were contributed by AF, YZ and JC. Draft of the manuscript was contributed by AF and YZ. Critical revision of the manuscript was contributed by WC. All authors contributed to the article and approved the submitted version.

## Conflict of interest

The authors declare that the research was conducted in the absence of any commercial or financial relationships that could be construed as a potential conflict of interest.

## Publisher’s note

All claims expressed in this article are solely those of the authors and do not necessarily represent those of their affiliated organizations, or those of the publisher, the editors and the reviewers. Any product that may be evaluated in this article, or claim that may be made by its manufacturer, is not guaranteed or endorsed by the publisher.

## Supplementary material

Supplementary Figure 1 LASSO Cox regression and construction of nomogram. **(A)** Coefficient profiles of variables in the LASSO Cox regression model. **(B)** Tenfold cross-validation for turning parameter selection in the LASSO Cox regression model. **(C)** Heatmap of 13 genes in the risk model in HS and LS groups. **(D)** The nomogram of the prediction model.

Supplementary Figure 2 Single-gene survival analysis of 13 genes in the prognostic model.**(A-M)** Single gene survival analysis of 13 genes (AFF3, B4GALNT4, CD38, CHRNA2, CST2, ADGRF5, KLK14, LRRC31, MT1F, MT1G, SFTPA2, SLC7A4, TDRD1) in prostate cancer.
