# Supplementary figures and images for: A novel prognostic model for prostate cancer based on androgen biosynthetic and catabolic pathways
Source: Front Oncol. 2022 Nov 10;12:950094. doi: 10.3389/fonc.2022.950094 (PMC9685527; doi:10.3389/fonc.2022.950094)

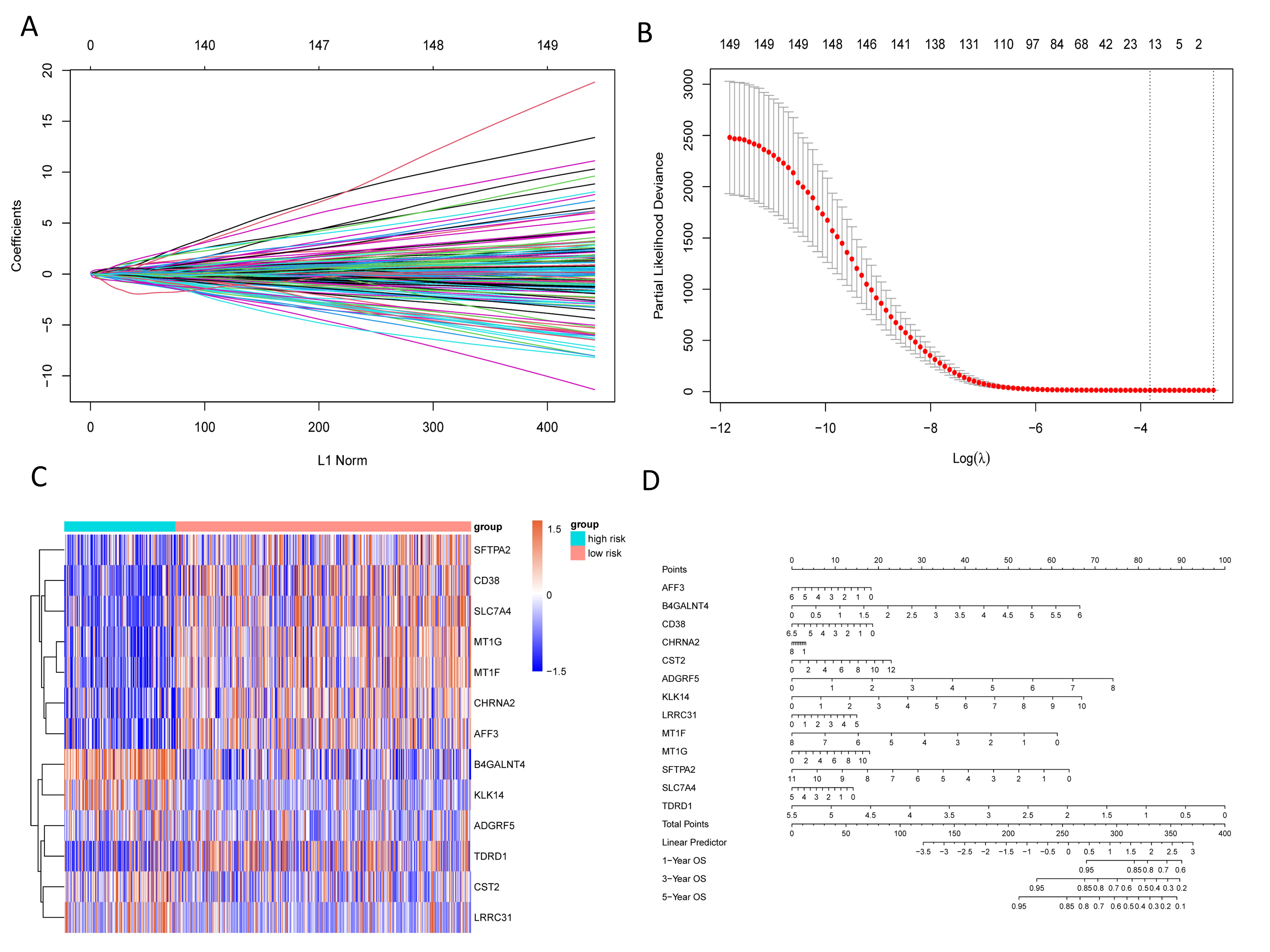

Supplement: Supplementary Figure 1 — LASSO Cox regression and construction of nomogram. (A) Coefficient profiles of variables in the LASSO Cox regression model. (B) Tenfold cross-validation for turning parameter selection in the LASSO Cox regression model. (C) Heatmap of 13 genes in the risk model in HS and LS groups. (D) The nomogram of the prediction model. [file Image_1.tif]

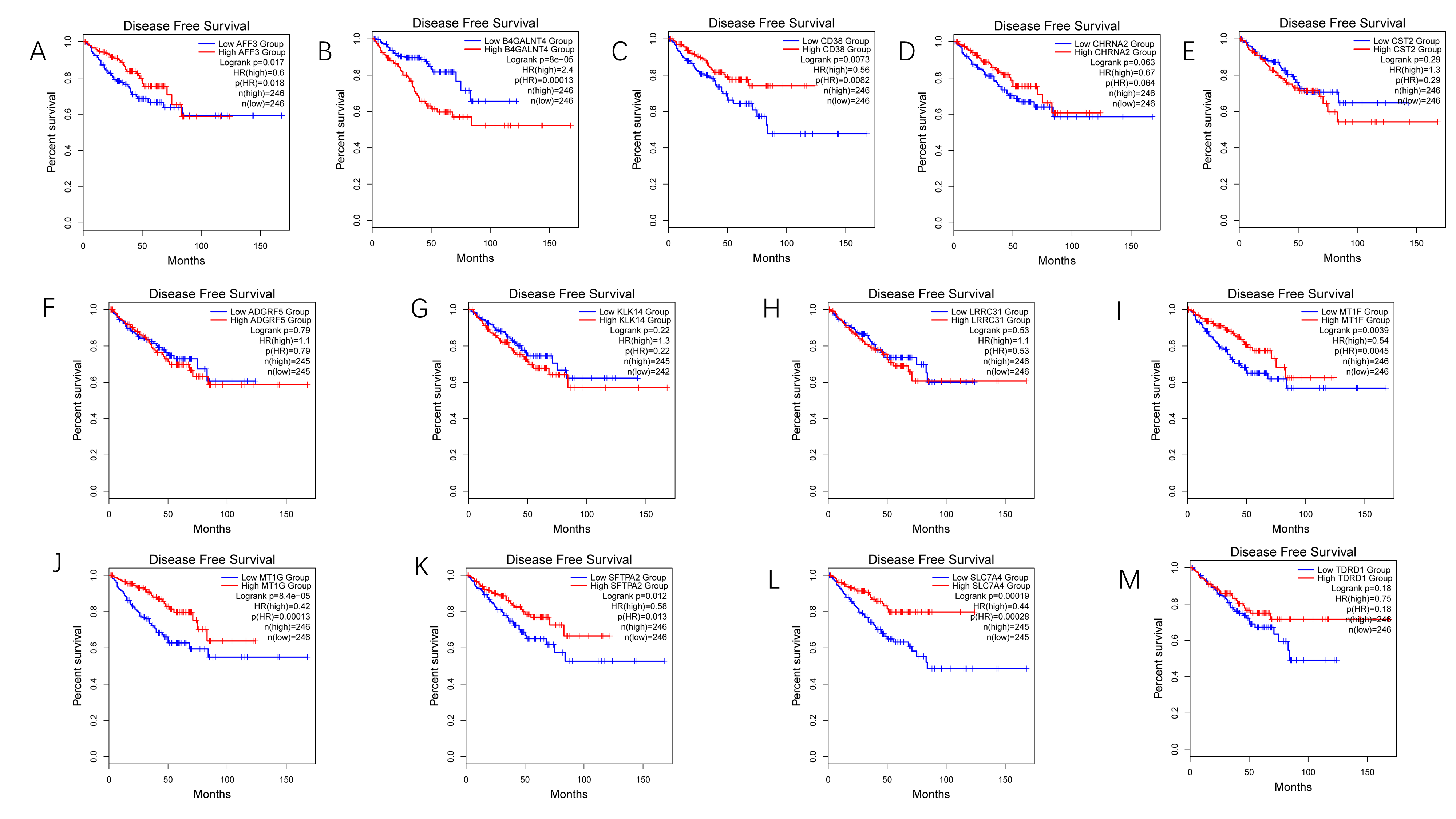

Supplement: Supplementary Figure 2 — Single-gene survival analysis of 13 genes in the prognostic model.(A-M) Single gene survival analysis of 13 genes (AFF3, B4GALNT4, CD38, CHRNA2, CST2, ADGRF5, KLK14, LRRC31, MT1F, MT1G, SFTPA2, SLC7A4, TDRD1) in prostate cancer. [file Image_2.tif]
