# Supplementary material for: A novel prognostic model for prostate cancer based on androgen biosynthetic and catabolic pathways
Source: Front Oncol. 2022 Nov 10;12:950094. doi: 10.3389/fonc.2022.950094 (PMC9685527; doi:10.3389/fonc.2022.950094)
Supplement: Supplementary file 3 [file Table_1.docx]

***Supplementary material1:*** *Summary of the cutoff value in the cohorts used in this study*

| **Cohort** | **Cutoff value** |
| --- | --- |
| TCGA-PRAD | 0.6855398 |
| MSKCC | -1.790379 |
| GSE70770 | -2.053357 |
